# Individual personality predicts social network assemblages in a colonial bird

**DOI:** 10.1038/s41598-023-29315-3

**Published:** 2023-03-01

**Authors:** Fionnuala R. McCully, Paul E. Rose

**Affiliations:** 1 School of Environmental Sciences, University of Liverpool, Liverpool, L3 5DA UK; 2 Centre for Research in Animal Behaviour, Psychology, University of Exeter, Exeter, Devon EX4 4QG UK; 3 WWT, Slimbridge Wetland Centre, Slimbridge, Gloucestershire GL2 7BT UK

**Keywords:** Animal behaviour, Behavioural ecology

## Abstract

Animal personalities manifest as consistent individual differences in the performance of specific behavioural expressions. Personality research has implications for zoo animal welfare, as it can further our understanding of how captive individuals may differ in their resource use and provide insight into improving individual and group social health. For group living species, personality may enable assortment based on similar behaviour and influence an individual’s interactions with conspecifics (e.g. social support). This research aimed to document how personality traits (aggressive, exploratory, submissive) influenced the social network structure of highly social animals in a captive environment. Data were collected from separate flocks of captive Caribbean (*Phoenicopterus ruber*) and Chilean flamingos (*Phoenicopterus chilensis*) to identify relationships between birds and examine opportunities for social support. The flocks associated non-randomly, and in both cases, personality was a substantial predictor of network structure. Personality also predicted key elements of Caribbean flamingo social role (degree, betweenness and average association strength) conflict outcome, and propensity to provide social support, however these patterns were not replicated within the Chilean flamingo network. While both species appear to assort by personality, the broader relationship between personality and social role may vary depending on species and context.

## Introduction

In group living species, animal personality (consistent differences in individual behaviour)^1,2^ is known to influence the relationship between an organism and its social environment^3,4^. As an individual’s personality may affect both affiliative^5^ and agonistic^6^ relationships, controlling for individual differences in behaviour may be critical for the effective study of gregarious species^7^. Although personality is generally stable over both time and context^8^, some studies report that when exposed to certain social stimuli, animals may exhibit behavioural plasticity^9^. An individual’s role within its social networks can be one such personality modifier- and whilst social role influences personality, personality also influences social role^10^.

The interplay between personality and social position drives a pattern noted in some hierarchical systems, where dominant positions are held by individuals with bold or outgoing personality traits (e.g. Refs.^11–13^). Certain behavioural strategies may prepare individuals for appropriate social roles^14^, however, experience and conspecific feedback can cause individuals to alter their behaviour, driving them to adopt more appropriate behavioural strategies within the immediate social environment^9^. Understanding the drivers of social role can inform captive management, as individual social behaviour potentially impacts upon social hierarchy and stability. Flack et al.^15^ showed that higher ranking pig-tailed macaques (*Macaca nemestrina)* facilitated social cohesion within captive groups and that their removal caused increased aggression, reduced cooperation and increased social clustering. This social distress subsided once the group had been restored, suggesting that the roles of keystone individuals must be understood if management strategies are to encourage internal social regulation.

Social Network Analysis (SNA) is commonly used to extract useful information on social roles^16^ and association patterns^4^ from group observations. SNA has revealed that animals across taxa invest time with preferred group members with whom they associate more often than by chance^17–19^. Social preference can be based on physical traits, for example choosing associates of a certain body length^19^, or based on personality. Some non-random associations accrue clear advantages, such as predator confusion^20^, but the adaptive value of associating by personality is more cryptic. Male great tits *(Parus major)* associate according to exploratory tendency^21^, while captive chimpanzees *(Pan troglodytes)* have companions with similar boldness tendencies as themselves^22^. This may make it easier for individuals to predict the behaviour of your social companions, which might be beneficial in situations where cooperation is required. For shy individuals, this technique may also be employed to avoid aggression. Shy three-spined sticklebacks *(Gasterosteus aculeatus)* maintained relatively few associates, but these strong relationships provided defence against bolder conspecifics^20^. Regardless of underlying mechanism, denying individuals access to their preferred social partners may negatively impact upon stress levels and group cooperation^23^, for example interfering with the provision of social support. Social support is defined as one individual providing another with aid, and this can take a number of different forms including assistance during agonistic encounters and consolidation^24^. Some species are known to provide social support of higher quality to individuals with which they maintain close affiliative relationships^25^, but the interaction between relationship strength, personality and the individual’s desire to provide social support requires further investigation.

Mapping the social networks is only the first step towards deeper analysis of zoo-housed populations^26^. Once obtained, these data have the potential to inform management strategies such as enclosure design^27^, tracking disease transmission^28^ and translocation^29^ all of which has the potential to impact individual and group wellbeing. Alternatively, SNA can be used to provide evidence of change following intervention^30^. Such monitoring is critical in captive populations, as the promotion of healthy social relationships are important if high-standard living conditions are to be maintained^26,31^. As there are many interaction types (e.g. aggressive or sexual) and numerous potential causes of associations^16^, supplementary information is often required for interpretation, and in captivity, data on influential factors such as personality can be used to evaluate individual network position. In such studies, including the present one, the term personality is applied to the individual differences in behaviour which are discernible in a group context, without direct intervention by the researcher to solicit a particular behavioural response (e.g. a test)^32–34^. Captive populations can be ideal subjects for personality research as the identification of individuals is often easily achieved in captive settings^10^ and the findings may have immediately actionable management and welfare applications^35–37^.

As many zoo-housed species are housed in large groups, understanding how personality influences characteristics of the social group, is important for gathering evidence for best practice management of captive populations. This study aimed to explore the impacts of personality on the social dynamics of separately housed flocks of captive Caribbean (*Phoenicopterus ruber*) and Chilean flamingos (*Phoenicopterus chilensis*). Flamingos are a suitable study species for this question because they are known to form strong, long lasting relationships with multiple individuals throughout the year^38,39^. Despite this, they also engage in agonistic interactions, with aggression being used during reproductive competition and resource acquisition^40^. Flamingos are sensitive to the relationship between social conditions and welfare. As this may influence the success of breeding programmes, information on their interactions has great potential for application^41^. This study’s aim was accomplished by (1) creating social networks and exploring patterns using SNA, (2) establishing how personality influences a flamingo’s association patterns and social role within its network, (3) investigating personality as a predictor of agonistic behaviour and the amount of social support given/received. It was anticipated that personality would predict social role and aggressive and exploratory birds would have more associations because they partook in activities that shyer birds avoided. It was predicted that less aggressive birds would have stronger associations and that these would be maintained with more frequent social support. It was also anticipated that bolder birds would win more agonistic encounters than shyer birds. Alongside personality, the effects of other factors such as sex, origin (wild caught or captive bred) and age were also considered within the wider context of the social network.

## Results

### Social network analysis

The flocks did not undergo direct statistical comparison, and so the results described below can only be applied to the focal social network. Both flocks were well connected, with high clustering coefficients (Caribbean = 0.853, Chilean = 0.820), thus there is a low level of cliquishness with the groups. The average path length was similar between the flocks (Caribbean *Mean* = 1.202, Chilean *Mean* = 1.223) indicating that (on average) a node required slightly more than one edge to reach any other node in the network. This points to a high level of connectivity between individuals. The average association strength in both flocks was 0.066, meaning that on average, individuals spend 6.6% of their time with another given individual within the flock overall.

**Figure 1:**
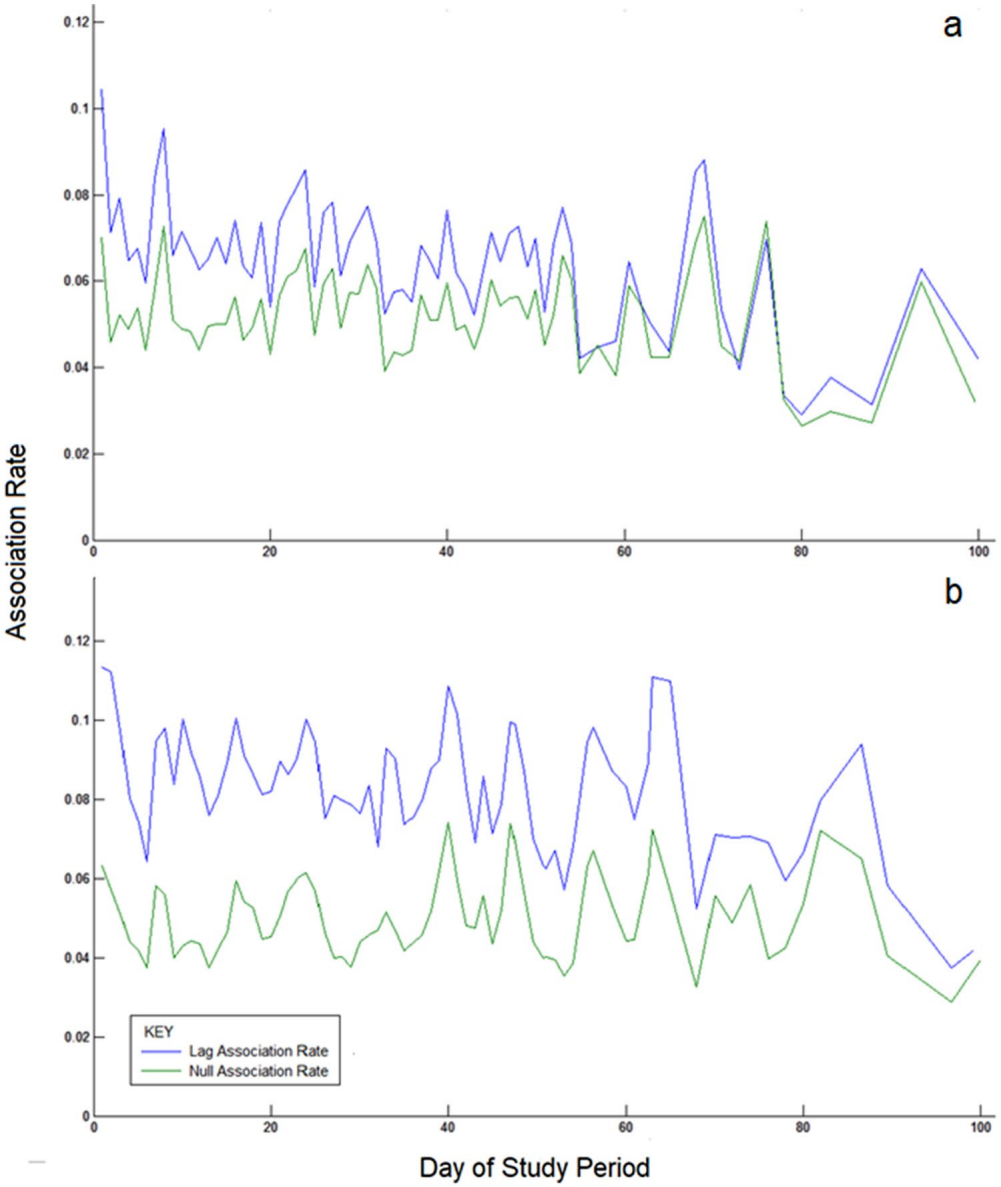
Comparison of the lag (blue) associations rates (the observed probability that two individuals which are associating during a particular sample session (day) will associate again in the future) and the null (green) associate rates (the expected lag association rate if the birds were randomly associating) for the Caribbean (**a**) and Chilean (**b**) flocks. In both cases, the lag association rates were statistically higher than the null associate rates (Caribbean, p = 0.026; Chilean, p < 0.001) suggesting non-random assortment.

In both flocks, temporal analysis provided evidence of non-random association, however this effect was much stronger in the Chilean flock than in the Caribbean flock. In the Caribbean flock, the lag rate (*Mean* = 0.07, *S.D* = 0.02) was significantly higher than the null rate (*Mean* = 0.06, *S.D* = 0.03) across the sample period (Fig. 1) with *t* (68) = 2.28, *p* = 0.026. The difference between the lag (*Mean* = 0.08, *S.D* = 0.01) and null (*Mean* = 0.05, *S.D* = 0.01) association rates was much more pronounced in the Chilean flock (Fig. 1) (*t* (63) = 24.20, *p* < 0.001). As the probability of two birds associating regularly was substantially higher than the expected probability if the birds associated by chance, it is therefore likely that both species were actively assorting with preferred individuals.

### Personality and association patterns

**Figure 2:**
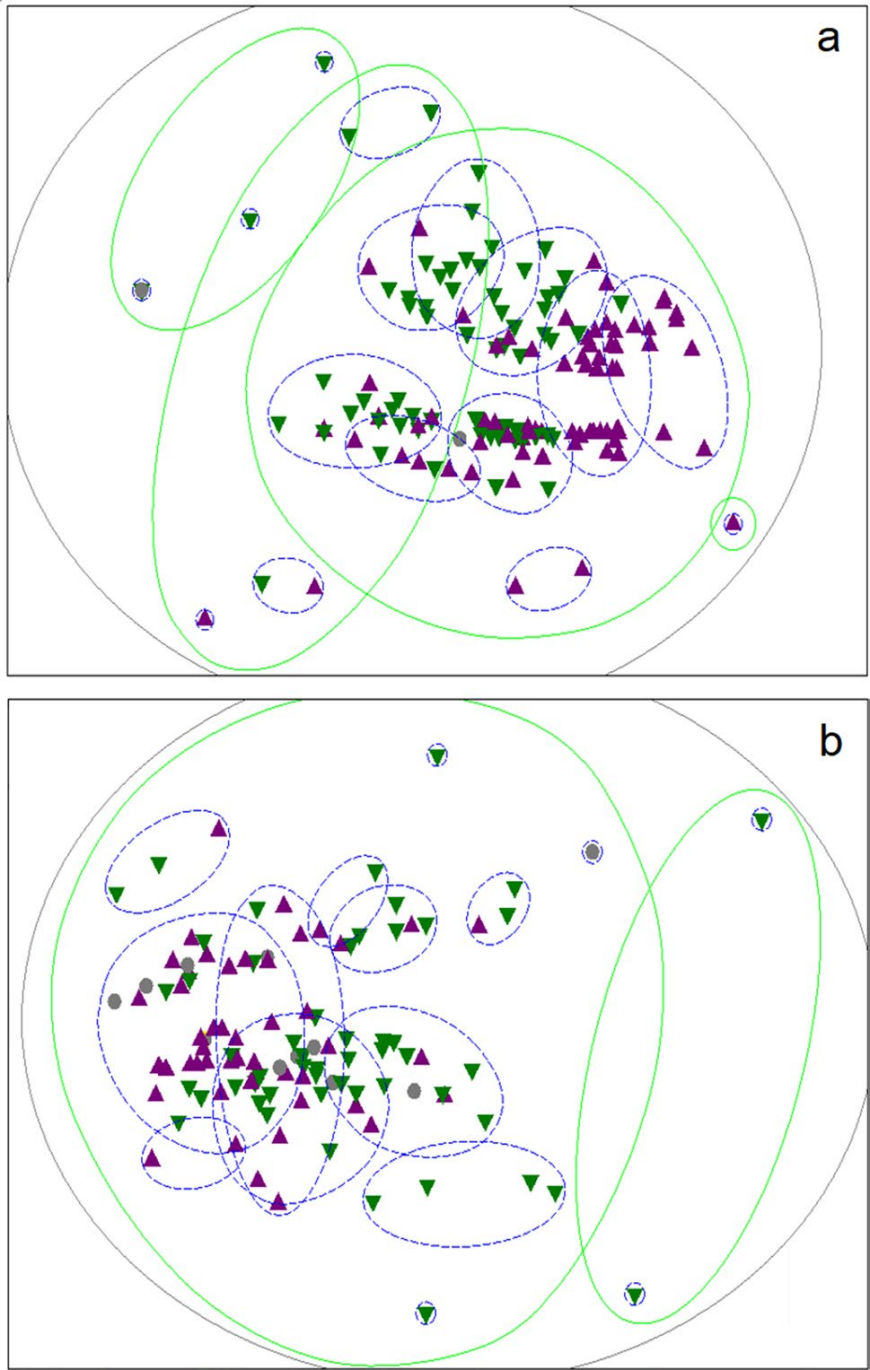
The Bray–Curtis similarity matrices which compared how similar individuals were in their behaviour (aggressive, submissive, exploratory) were visualised for both the Caribbean (**a**) (2D stress = 0.14) and Chilean (**b**) (2D stress = 0.15) using non-metric multidimensional scaling (NMDS) using PRIMER 6 ^68^. This plotted each bird’s score in a 2D space and grouped them according to similarity. Birds which expressed similar behavioural patterns are located closer to each other on the graph. Hierarchical cluster analysis was applied to generate percentage threshold of similarity. All the Caribbean birds were at least 34% similar in their behaviour, while all the Chilean birds were at least 29% similar in their behaviour (represented by the outermost grey circle in each panel respectively). The 50% (green, solid circles) and 80% (blue, dashed circles) similarity thresholds are displayed to better identify sub-groups of birds which display increasingly similar behavioural patterns. Male (purple, ▲), female (green, ▼) and birds of unknown sex (grey, ●) are distinguishable by colour and/or shape.

Non-metric multidimensional scaling (NMDS) showed that, when compared to one another all members of the Caribbean flock had a minimum behavioural similarity of 34%, while all members of the Chilean flock were at least 29% similar to one another (Fig. 2). Therefore, the behaviour of the Caribbean flamingos tended to be more homogenous, when compared to the Chilean flamingos.

For each flock, a single component was extracted during PCA (Table 1). The Caribbean component accounted for 54.54% of the variance within the data, while the Chilean component accounted for 49.48% (Table 1). Based on the positive factor loadings extracted from the PCA which condensed the personality variables, individuals from both species which were allocated high personality score engaged in more aggressive, exploratory and submissive behaviours than birds with lower personality scores (Table 1).

**Table 1:** Extraction results of the PCA of the three personality traits, aggressive, exploratory and submissive, for the separate flocks.

	Component 1	
	Caribbean	Chilean
Eigenvalue	1.64	1.48
% Variance explained	54.54	49.48
Aggressive	0.61	0.61
Exploratory	0.44	0.37
Submissive	0.65	0.69

Multiple Regression Quadratic Assignment Procedure (MRQAP) tests for both flocks showed that personality score category (see methods), sex and origin were significant predictors of the association matrix. In both flocks, individuals associated with conspecifics with a similar personality score to themselves (Caribbean; personality (controlling for other predictors) *partial correlation* = − 0.055, *p* = 0.014. Chilean; personality (controlling for other predictors) *partial correlation* = − 0.044, *p* = 0.024). For both flocks, sex (Caribbean *partial correlation* = − 0.034, *p* = 0.029; Chilean partial correlation = 0.028, *p* = 0.014) and origin (Caribbean partial correlation = 0.084, *p* = 0.004; Chilean partial correlation = − 0.044, *p* = 0.015) were also significant predictors of associations. In contrast, age was only a significant predictor for the Caribbean associations (*partial correlation* = − 0.153, *p* < 0.001) and was not significant for the Chilean associations (*partial correlation* = − 0.014, *p* = 0.497).

### Personality, social role and social support

The importance of personality as a predictor of social role and social support varied between species. In the Caribbean flock, three indicators of social role were successfully predicted by personality, although the overall models had relatively low explanatory power (Table 2). With each unit increase in personality score, node degree increased by 4.84 edges (R^2^ = 0.09, *F* = (1, 145) 8.43, *p* =  < 0.001; *b* = 4.84, *p* =  < 0.001), meaning that birds which were more aggressive, exploratory and submissive had more network connections. Personality also explained 11% of the variance in average association strength (R^2^ = 0.11, *F* = (1, 145) 10.20, *p* =  < 0.001; *b* = 0.004, *p* =  < 0.001), meaning that birds with higher personality scores tend to form stronger bonds. The relationship between personality score and betweenness was the weakest of the three measures (R^2^ = 0.02, *F* = (1, 145) 3.23, *p* = 0.057; *b* = 0.69, *p* = 0.044), however these results indicate that birds with higher personality scores are more likely to link less closely connected network members when compared with less aggressive, less exploratory and less submissive individuals.

**Table 2:** Figures indicating individual model fit and predictor variable (personality, age) significance when modelled against measures of social role (node degree, betweenness and average association strength) and social support (total number of fights, total number won and lost, social support frequency) in the linear regressions with 1000 random permutation trials.

Species	Response variable	Model fit			Regression coefficient			
		R^2^	*F* value	*p* value	Personality score		Age	
					*b*	*p* value	*b*	*p* value
Caribbean	Social role							
	Node degree	0.09	8.43	< 0.001	4.84	< 0.001	− 0.24	0.969
	Node betweenness	0.02	3.23	0.057	0.69	0.044	− 0.07	0.976
	Average association strength	0.11	10.20	< 0.001	0.004	< 0.001	− 0.0020	0.968
	Social support							
	Total no. fights	0.42	55.17	< 0.001	3.11	< 0.001	− 0.02	0.727
	No. fights won	0.34	39.88	< 0.001	0.92	< 0.001	− 0.02	0.937
	No. fights lost	0.22	21.58	< 0.001	0.62	< 0.001	− 0.01	0.857
	Social support frequency	0.25	25.30	< 0.001	0.89	< 0.001	0.05	0.352
Chilean	Social role							
	Node degree	0.03	3.05	0.061	− 2.47	0.989	− 0.10	0.882
	Node betweenness	0.02	2.68	0.061	− 0.83	0.989	0.01	0.421
	Average association strength	0.01	2.33	0.120	0.00	0.973	− 0.00004	0.667
	Social support							
	Total no. fights	0.32	28.91	< 0.001	− 4.35	> 0.999	0.01	0.388
	No. fights won	0.17	13.29	< 0.001	− 1.78	> 0.999	0.03	0.170
	No. fights lost	0.37	35.16	< 0.001	− 1.45	> 0.999	0.0009	0.047
	Social support frequency	0.11	8.27	0.002	− 1.16	> 0.999	− 0.04	0.952

These results contrast sharply with the Chilean flamingo models, in which personality score could not explain individual variation in degree, betweenness or average association strength; although, once again, the explanatory power of these models was found to be very low. In both flocks, age was not associated with any of the social role metrics (Table 2).

A similar between-flock pattern to the one discovered for social role metrics was found in the models concerning conflict outcome (“fights”) and social support (Table 2). In the Caribbean flock, birds with higher personality scores were found to take part in more fights, (R^2^ = 0.42, *F* = (1, 115) 55.77, *p* =  < 0.001; *b* = 0.31, *p* =  < 0.001), meaning that these birds were found to both win (R^2^ = 0.34, *F* = (1, 115) 39.88, *p* =  < 0.001; *b* = 0.92, *p* =  < 0.001) and lose more fights than conspecifics with lower personality scores (R^2^ = 0.22, *F* = (1, 145) 35.16, *p* =  < 0.001; *b* = , *p* =  > 0.999). Again, no such patterns were found within the Chilean data, and so there was no evidence that personality score had an impact on conflict outcome in this species (Table 2).

Similarly, personality score was found to increase the frequency of social support provided by the Caribbean flamingos (R^2^ = 0.25, *F* = (1, 145) 25.30, *p* =  < 0.001; *b* = 0.89, *p* =  < 0.001), but not the Chilean flamingos, where it had no effect (R^2^ = 0.11, *F* = (1, 115) 8.27, *p* = 0.002; *b* = − 1.16, *p* =  > 0.999). Age was found to be a non-significant influence on conflict outcome and social support in both species, save for one result where older Chilean flamingos may be more prone to losing fights when compared to younger flock mates (R^2^ = 0.37, *F* = (1, 115) 35.16, *p* =  < 0.001; *b* = 0.0009, *p* = 0.047).

## Discussion

By applying SNA to quantify the relationships of Caribbean and Chilean flamingos in captivity, we are able to describe two well-connected and actively assorting flocks (Fig. 1) which nevertheless differ in their specific drivers of social behaviour. Both flocks demonstrate variation in individual behavioural traits (i.e. personality) (Fig. 2) and appear to use this to determine association choices. Although the flocks were not compared inferentially, we detected distinct species-specific differences in the importance of both individual personality and age as a predictor of social role and agonistic behaviours. In the Caribbean flock, personality was found to have an effect on social role, in that individuals which displayed higher levels of aggressive, exploratory and submissive behaviour had more numerous and stronger network connections. Such birds were also more frequently observed engaging in fights, and appeared to be more willing to provide social support when network associates were threatened (Table 2). In contrast, the personality of the Chilean flamingos did not affect their social role metrics or agonistic behaviour, however ‘losing’ fights was a more commonly recorded outcome for older birds. The evidence suggests that age played a different role in the Caribbean flock, where it influenced association patterns but not social role, fight frequency/outcome or social support.

The present study supports previous findings that flamingos have preferred social partners^39,40^ and suggests that these associations might be influenced by an individual’s intrinsic traits. In both species, individuals were more likely to associate with birds which were similar in personality to themselves. Massen and Koski^22^ suggested chimpanzees surround themselves with predictable companions that will be more reliable if cooperation or altruism is required. Associating with similar conspecifics could incur similar benefits for flamingos, for example, as a strategy for avoiding unexpected aggression or, in the case of the Caribbean flock, to promote social support. Alternatively, exploratory and aggressive birds may associate when involved in competitive activity, however further observation would be required to confirm any of these interpretations.

The link between personality and association choice may also be related to reproductive success. In most cases, the strongest link displayed by an individual flamingo is that which it shares with its reproductive partner^42^ and it is possible that choosing a partner which is similar to oneself has implications for breeding success^43,44^. However, a study directed specifically towards this question would be required to explore this idea further.

Contrary to our predictions, personality only impacted social role in one of the study species (the Caribbean flamingos) and in places, its influence had the opposite effect to what was expected. Furthermore, given the low explanatory power of these models, these results should be interpreted with caution. As predicted, birds with a high personality score had a higher node degree, possibly because outgoing and aggressive tendencies predispose birds to engage in a wider range of activities (such as exploring and fighting), thereby associating with more individuals^4^. Conversely to our predictions, higher personality scores were also associated with stronger associations. Establishing strong relationships has previously been connected to interaction intervention^24^, thus if aggressive individuals are to engage in agnostic interactions more frequently, stronger network ties may facilitate the solicitation of social support from their close associates.

Caribbean flamingos with higher personality scores took part in more fights, leading to an increased frequency of both winning and losing for these individuals. A link between aggression and altercation frequency has also been reported in dark-eyed juncos (*Junco hyemalis oreganus*) and it is suspected to be connected to the evaluation of risk immediately before engagement^45^. Positive relationships between exploration and risk tolerance have been documented in other species, in that some individuals may be more willing to risk physical harm or additional energy expenditure in exchange for foraging or breeding opportunities^46,47^. In flamingo flocks, aggression is largely concerned with resource and reproductive competition^48^, with one study reporting that 70% of fights were won by the instigators^49^. Taken together, this evidence suggests that flamingos make decisions based on the perceived likelihood of victory. It is therefore possible that birds with higher personality scores are less averse to the potential negative outcomes of fights, and thus participate in these interactions more frequently. This can also be linked to our finding that birds with higher personality scores provide more social support in the Caribbean flock. Providing social support is not risk free, as helpers can often be targeted by aggressors^25^. Greater risk tolerance among individuals with higher personality scores may make them more willing to participate in fights.

The relationship between aggressive and exploratory behaviour has been highlighted before (e.g. Ref.^50^) however a positive correlation between these and submissive behaviour is rarer. A higher number of submissive behaviours may be a by-product of participating in more fights than shier individuals, or alternatively, submissive birds may be attacked more often^49^ causing them to engage in aggressive behaviours for defence. In future, separating submissive behaviour associated with fights from other submissive behaviours may produce a clearer picture.

A number of interspecies differences in the drivers of association patterns, social role and social support provision were noted as part of our analysis. While age was found to impact the association patterns of the Caribbean flamingos, it did not appear to affect the Chilean flamingos’ choice of social companions. Affiliative behaviour is influenced by age in a number of other species (e.g. Refs.^17,51^). The adaptive value of this behaviour may relate to familiarity^52^, which allows social partners to benefit by staying together over longer periods of time, thereby aging alongside one another. As the birds were also shown to associate according to origin (wild caught or captive bred), this demonstrates that this species can maintain associations over extremely long periods of time (decades), which may cause the relationship between age and association to develop passively^42^.

Unfortunately, the present study cannot answer the question of why one species’ interactions seem to be more heavily influenced by personality or age than the other. The Chilean flock was smaller than the Caribbean flock, and it has been shown that variation in group size can impact upon hierarchy and aggression in captive animals^53^. Chilean flamingos also breed later in the summer than the Caribbean flamingos^54^, and as breeding period has been shown to affect flock structure^48^, this makes it more challenging to directly compare the networks. Crucially, these results reiterate the message that conducting replicate studies is recommended before using SNA to generalise beyond the focal social network^55^. Ultimately, comparative studies regarding flamingo ecology are needed to establish if there are different selection pressures affecting social structure in Caribbean and Chilean flamingos. Wilson et al.^56^ lamented that SNA is not commonly used to investigate the adaptive benefits and ecological implications of social structure. Any progress in this field would retrospectively benefit the present study, as accounting for subtle differences in their ecology may explain the observed differences between the Caribbean and Chilean flamingos.

Considering SNA results and personality data in tandem has the potential to inform captive management strategies. The identification of highly aggressive individuals noteworthy, as abnormal aggression may require management in captivity^57^. In flamingos, such behaviour may result in egg damage and injury^41^. Furthermore, as the translocation of animals between collections can benefit for flamingo reproductive success^58^, SNA can be used to predict resulting changes to the flock’s stability. As flamingos have preferred social partners, translocation decisions should be made extremely carefully.

The present study provided a glimpse into the complex nature of social structure in captive animals by confirming that different behavioural types exist with captive Caribbean and Chilean flamingo flocks and by revealing that aspects of both species’ group dynamics, including association number and strength, are structured around this feature. Crucially, personality also appears to influence patterns of preferred association, suggesting that this factor plays an active role in the social lives of flamingos, even if that role varies somewhat between species. Furthermore, this study provides evidence that it is unwise to generalise beyond study species, even between members of the same genus. The lack of evidence that Chilean flamingo social role or social support are connected to personality acts as a reminder that differences in social structure can occur between species or even between networks. Comprehensive knowledge of the group in question is therefore essential when conducting studies of this nature. Finally, given that relationships between flamingos are often long-lasting, it is recommended that managers aim to keep established relationships intact when translocating birds between collections. The strategic removal of highly aggressive individuals can also be considered, especially if these birds are disruptive to breeding proggrammes. However, such a course of action should be taken after considering the role that aggression plays in everyday interactions for these species of bird.

## Methods

### Study population

Data were collected from separately housed flocks of Caribbean and Chilean flamingos between March and July 2014 at the Wildfowl and Wetlands Trust (WWT) Slimbridge Wetland Centre, Gloucestershire, UK. The Caribbean flock contained 134 individuals before 13 birds were added in May, bringing the total to 147. The number of Chileans remained consistent at 115. All birds were identifiable by unique Darvic leg rings. Both species were fed a complete flamingo pellet and had ad libitum access to indoor and outdoor spaces, all of which were visible to the public. At the time of data collection, there were no barriers between the birds and the public viewing points in the outdoor enclosures. Ethical approval was obtained from the University of Exeter's Psychology Ethics Committee and the Animal Welfare & Ethics and Welfare Committee at WWT Slimbridge. No ethical concerns were identified by either body.

### Collection of association data

As the two species were housed separately, two complete datasets and social networks were produced; one per species. The data collection methods were identical between the two flocks. The data collection methods applied in this study are similar to those outlined in previous literature on flamingo social networks^39,40,42^. The birds had ad libitum access to their whole enclosure and so could choose to mix with preferred conspecifics. High quality photographs were taken four times a day (at 10:00, 12:00, 15:00 and 16:30) four consecutive days a week^39^ (Appendix 1). Individuals were identified in the photographs by their Darvic rings, and singles, pairs and groups were recorded. Birds were considered to be associating if they were within one neck length of each other^26^. The ‘chain rule’ was also applied to define associations between birds which had formed smaller subgroups (two or more birds standing more than one neck length away from the adjacent subgroup) within the main flock^39^. Birds within the same subgroup but outside the threshold distance of one neck length were still considered to be associating through other subgroup members^16^. The frequency of these proximity-based interactions between individuals formed the association data upon which the social networks were based^26^. This method, known as ‘the gambit of the group’, infers association through group membership^59^ and was used to quantify the association strength between individuals (i.e. birds seen together more often were considered to have a stronger association). Although such methods can artificially inflate association number and strength^60^, with repeated sampling it is suitable for studies of closed groups, whose members form short-term aggregations and rest in close proximity to one another^61^. During photograph analysis, if an individual’s identity remained unverified, it was not included in that particular sample. If an unidentified bird was standing in a pair, the other bird was also discounted. In groups of more than two, unidentified birds were excluded, while associations between identifiable birds were recorded.

### Social network analysis

The construction and analysis of the social networks were carried out using the computer programmes SOCPROG 2.4 (Whitehead, 2009), UCINET 6^62^ and NetDraw 2.062^63^. Via SOCPROG, the list of associations for each flock was translated into a matrix of symmetric association indices, allocating a weighting between 0 (never seen together) and 1 (always seen together) to each association based on frequency of occurrence. Identification of all individuals within a sample is difficult in photograph evidence collected from large numbers of animals which are spatially concentrated^64^ (such as captive flamingo flocks). Furthermore, flamingos regularly hide their Darvic rings by standing on one leg^42^, leading to higher rates of concealed associate identity. To control for this bias, a half-weight association index (HWI) was applied to these data as it is thought to be the most reliable way of calculating association indices when universal identification of the group is impossible in any given sample^64,65^. The association matrixes were then transplanted into UCINET, and then into NetDraw, which created social networks based on the varying association strengths, from which the metrics of social role were derived. These were node degree (the number of edges joined to a node), node betweenness (the number of shortest paths between nodes which pass through the focal node) and average association strength (average association index for each individual based on matrix of symmetric association indices, where birds with higher scores tended to spend more time with specific individuals). These were chosen based on their use in other zoo-housed studies (e.g. Refs.^30,31^) and to facilitate comparison with other SNA research conducted on flamingos^42^.

### Collection of personality data

In the captive environment, personality is commonly measured through the behavioural observation of individuals within the group setting (e.g. Refs.^33,34^), as opposed to through the use of testing protocols often applied in situ^32^. Personality data were collected through the event sampling of individuals over an extended period and multiple contexts in a non-experimental setting, and the frequency of behaviours know to be connected to specific personality traits were recorded^32^. Known behaviours were connected to the personality traits aggressive, exploratory and submissive (Appendix 2). These traits were chosen as aggressive birds are known to be problematic during nesting in zoo-housed flocks^41^, while submissive and exploratory behaviours are regularly witnessed during observation, but have yet to be tested empirically (Rose. P. E, personal observation). They were represented by continuous axes and were assessed through distinctive and common behaviours. Observation sessions consisted of four-hour event sampling periods, during which each instance of a relevant behaviour was recorded alongside bird identity. Birds could be aggressive, submissive and/or exploratory within the same observation period and each instance was recorded. Eight hours of data, composed of one morning and one afternoon session, were collected per species, per week (Appendix 1).

### Sex, age and origin data

Sexing data (male, female, unknown), origin information (wild caught or captive bred) and actual (captive bred) or estimated (wild caught) ages were extracted from species360^©^ Zoological Information Management System (ZIMS) provided by the WWT.

### Collection of social support data

**Figure 3:**
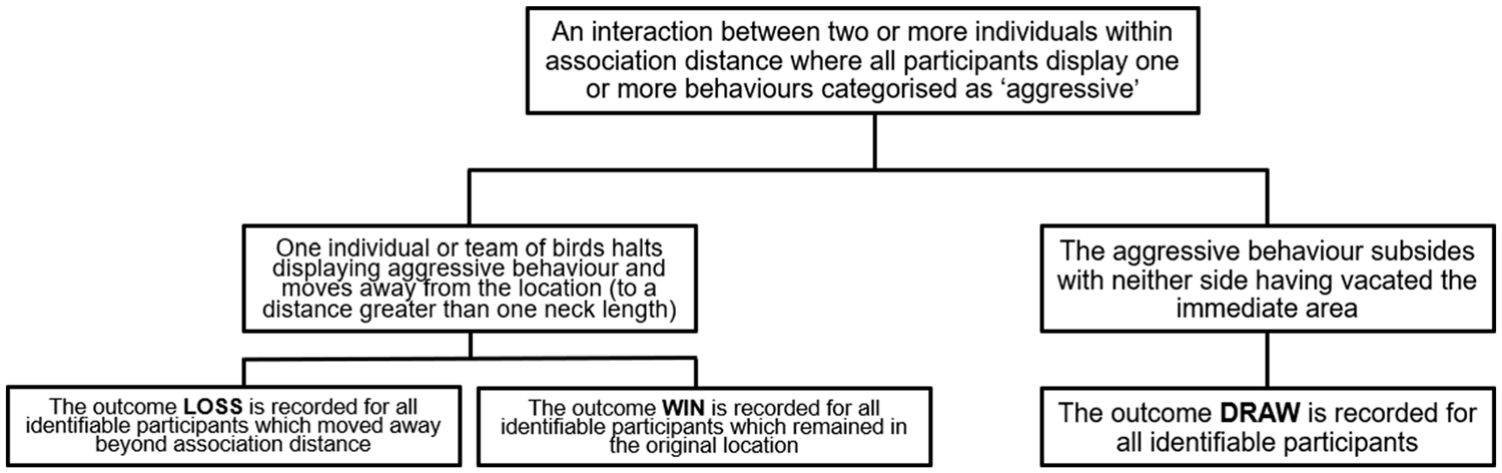
Criteria for the recording of fight outcome in the collection of social support data.

Affiliative behaviours are rare in flamingos; an absence of aggression can be used as a proxy^40^. In comparison, aggression between individuals is extremely common within colonies^66^, therefore the present study focuses on social support provided during agonistic interactions. A fight was defined as an interaction between two or more individuals standing within association distance (one neck length), where all participants display one or more behaviours categorised as ‘aggressive’ (see supplementary material). Observation sessions consisted of three-hour event sampling periods (divided evenly between morning and afternoon sessions) amounting to six hours of data per flock per week (Appendix 1). The identity of each bird involved in the interaction was recorded alongside their outcome in the fight. This was recorded as loss, win or draw (Fig. 3). A bird was providing social support if it was engaged in aggressive behaviour alongside another bird (within one neck length) against a common opponent, but did not instigate the interaction. To be counted, following the receipt of the social support, the focal birds could not be displaced or forced to retreat by the interaction, thereby suggesting some benefit of this behaviour. In the event that one or more birds remained unidentified, the data were recorded for those birds which could be identified.

### Data analysis

Temporal analyses were conducted using SOCPROG to test if the flamingos were associating randomly. Lagged associations rates (the observed probability that two individuals which are associating during a particular sample session (day) will associate again in the future) were compared to null associate rates (the expected lag association rate if the birds were randomly associating) with paired-sample t-tests using IBM SPSS Statistics 21.0^67^.

The personality data were tallied so that each bird was assigned three separate scores; aggressive, exploratory and submissive. Each individual’s tallied scores for these three behavioural traits were used to create a Bray–Curtis similarity matrix which quantified how similar birds were in their behaviour. To help explore the trends of behavioural similarity within the two flocks, this matrix was visualised alongside sex using non-metric multidimensional scaling (NMDS) using the PRIMER 6 software^68^, which plotted each bird’s similarity score in 2D space and grouped them according to similarity, where birds in closer proximity expressed similar behavioural patterns (Fig. 2). The three personality variables were found to be positively correlated in both flocks (Appendix 3). Using the *factoextra* package^69^ in R^70^, principle component analysis (PCA) was applied to condense the three behavioural tallies into a single score to account for multicollinearity^71^ and to avoid running individual analyses which would lead to type 1 error inflation. Each flock’s data were analysed in a separate PCA to produce a single personality score for each individual. To determine the contribution of specific predictors (personality score, age in years, and separate binary variables representing sex and origin- wild or captive bred- of each bird) as explanatory variables of the association matrix, Multiple Regression Quadratic Assignment Procedure (MRQAP) testing was conducted in Socprog for each flock^72,73^. Personality scores were grouped into categories to enable similarity in personality score to be tested within the matrix. Personality scores were categorised as: A < − 0.9; B − 0.89 to 0; C 0.1 to + 1.0; D > 1.1, where larger scores were indicative of more frequent aggressive, exploratory and submissive behaviours. As sex^18^ and age^17^ might also affect association preference, we controlled for these variables in the association analyses. We also controlled for each individual’s origin (wild caught or captive bred) in this test. The association matrix was used as the dependent variable. Permutations were run from 1000 to 10,000 to see the stability of *p* values and partial correlation (r values).

The social role and social support data were processed using linear regression in UCINET (to account for a lack of independence) which calculated 1000 random permutation trials to create a sampling distribution from which the correct standard errors were extracted^74^. Each flock was analysed separately using the personality score as the predictor variable. In UCINET, seven response variables were analysed in individual models. Three of these response variables represented social support: total number of fights, number of fights won and lost, and social support frequency. The remaining four response variables represented social role: node degree, node betweenness and average association strength. Personality score was used as the predictor variable in all models.

We stress that the two social networks were not directly compared through the use of inferential statistics. Thus, the patterns described in the results refer only to the specific network under discussion, and these may not be found in other networks of the same species.

## Supplementary Information

Supplementary Information.

## Data Availability

The datasets used and analysed during the current study available from the corresponding author on reasonable request.
