# Supplementary material for: Individual personality predicts social network assemblages in a colonial bird
Source: Sci Rep. 2023 Mar 1;13:2258. doi: 10.1038/s41598-023-29315-3 (PMC9977762; doi:10.1038/s41598-023-29315-3)
Supplement: Supplementary file 1 — Supplementary Information. [file 41598_2023_29315_MOESM1_ESM.pdf]

Individual personality traits predict social network assemblages in a colonial bird- supplementary material

F. R. McCully & P. E Rose

Appendix 1: Example weekly timeline of data collection

Data were collected on four consecutive days a week between March and July 2014. Although these days were always consecutive, the exact days during which data collection occurred varied week on week. Social network photographs were taken of both flocks every day at 10:00, 12:00, 15:00 and 16:30. Personality data collection sessions lasted four hours. One morning session (09:00-13:00) and one afternoon session (13:00-17:00) was conducted per flock per week. Social support sessions lasted three hours. One morning session (09:00-12:00) and one afternoon session (14:00-17:00) was conducted per flock per week. Where possible one personality session and one social support session was conducted per day to allow time to recover from observer fatigue. To minimise disturbance to the flocks, if data were collected from the Chilean birds in the morning, the afternoon would be allocated to the Caribbean flock if possible.

Supplementary Table A1: A timeline of a hypothetical week, demonstrated how all the data collection sessions might have occurred during the study period. Social network photograph times are represented by the vertical, red, dashed lines. Caribbean (shades of green) and Chilean data collection sessions (shades of pink) are individually labelled.

<table>
<tr><th rowspan="2"></th><th colspan="9">Time of Day</th></tr>
<tr><th>09:00</th><th>10:00</th><th>11:00</th><th>12:00</th><th>13:00</th><th>14:00</th><th>15:00</th><th>16:00</th><th>17:00</th></tr>
<tr><td>Mon</td><td colspan="9">No Data Collection</td></tr>
<tr><td>Tue</td><td colspan="4">Personality: Caribbean</td><td></td><td colspan="3">Social Support: Chilean</td><td></td></tr>
<tr><td>Wed</td><td colspan="3">Social Support: Caribbean</td><td></td><td colspan="4">Personality: Chilean</td><td></td></tr>
<tr><td>Thu</td><td colspan="4">Personality: Chilean</td><td></td><td colspan="3">Social Support: Caribbean</td><td></td></tr>
<tr><td>Fri</td><td colspan="3">Social Support: Chilean</td><td></td><td colspan="4">Personality: Caribbean</td><td></td></tr>
<tr><td>Sat</td><td colspan="9">No Data Collection</td></tr>
<tr><td>Sun</td><td colspan="9">No Data Collection</td></tr>
</table>

Appendix 2: Ethogram of behaviours

Supplementary Table A2: Ethogram used to categorise behaviours related to aggressive, outgoing and submissive personality traits

Personality Trait	Behaviour Name	Description
**Aggressive**	Non-Contact Aggression	While remaining stationary, two or more individuals extend their necks towards each other, beaks open without contact.
	Contact Aggression	While remaining stationary, one individual moves its head rapidly toward another so its beak contacts its body, neck or head.
	"Chrysanthemuming"	Scapular feathers are raised and spread apart. Can be whilst stationary or travelling.
	Head Shake	While remaining stationary, head is swayed from side to side, beak open in the direction of a conspecific.
	Stand on Conspecific	A standing individual steps onto the back of an individual resting on the ground. Standing individual's foot may be raised and lowered onto the resting individual's back and/or neck one or more times.
	Displacing	Whilst moving between locations, one individual approaches another, applies directed physical contact with its beak, causing the other individual to move away from its previous location.
	Push	Whilst moving between locations, one individual approaches another, applies directed physical contact with its body, causing the other individual to move away from its previous location.
	Interspecies Aggression	An individual displays a listed aggressive behaviour towards another species within the exhibit (e.g. a crow or duck).
**Submissive**	Submission	Whilst in a stationary location, an individual is approached by a travelling conspecific and receives physical contact (see 'push' and 'displacing' for examples). The previously stationary individual moves rapidly away from the conspecific, feathers are kept flat against the body, no vocalisation.
	Receives Conspecific Aggression	An individual is the subject of an aggressive behaviour from a conspecific (e.g. 'contact aggression' or 'stand on conspecific') but does not exhibit aggressive behaviours during the interaction . Remains stationary.
	Receives Interspecific Aggression	An individual is the subject of an aggressive behaviour from another species (e.g. crow or duck) but does not exhibit aggressive behaviours during the interaction. May remain stationary or move location.
	Follow	An individual walks between sections of its enclosure whilst being led (see 'lead') by another individual within association distance (one neck length).
**Exploratory**	Enters New Area	An individual walks alone into a section of the enclosure (at least ten metres from the nearest conspecific) which has devoid of any other conspecifics since the start of the observation session.
	Lead	An individual walks between sections of its enclosure whilst being followed by at least one other individual (see 'follow') within association distance (one neck length).
	Return After Disturbance	The first individual to return (alone or followed by others, see 'lead') to an area recently disturbed by humans

Appendix 3: Correlations of personality variables

The three personality variables (aggressive, exploratory and submissive) were tested to detect any correlations (Man Whitney U test) within the data which might lead to multicollinearity in the models. All relationships were significantly positively correlated (Table A3).

Supplementary Table A3: Results of the Man Whitney U correlation tests conducted on the personality variables. In all cases the test statistic (W) is presented alongside an indicator of significance (based on p-value). *p = < 0.001

		Aggressive	Exploratory
Caribbean	Exploratory	1564*	-
	Submissive	4500.5*	3915.5*
Chileans	Exploratory	534.4*	-
	Submissive	1619*	2564*
